# Stress-Induced Dysregulation of Brain Iron Metabolism and Its Links to Neurological Disorders

**DOI:** 10.3390/biology14111575

**Published:** 2025-11-11

**Authors:** Ke Dong, Bing Liu, Gang Cheng, Yang Li, Fang Xie, Jianning Zhang, Lingjia Qian

**Affiliations:** 1 Beijing Institute of Basic Medical Sciences, Beijing 100850, China; dongke0302@163.com (K.D.); liubingzsp@163.com (B.L.); 13201210710@163.com (Y.L.); vancoxie1@163.com (F.X.); 2 Department of Neurosurgery, First Medical Center of the PLA General Hospital, Beijing 100853, China; yjscg2003@126.com; 3 School of Medicine, South China University of Technology, Guangzhou 511442, China

**Keywords:** stress, iron metabolism disorders, iron-chelation, neurological diseases

## Abstract

Stress is a non-specific systemic response to internal or external challenges. Recent studies show that stress can disrupt iron metabolism and that iron dyshomeostasis is implicated in many diseases-particularly within the nervous system, where iron distribution and regulation intersect tightly with oxidative stress and inflammation. Activation of the hypothalamic–pituitary–adrenal (HPA) axis by stress can upregulate hepatic hepcidin and reprogram systemic iron fluxes, leading to functional iron deficiency and, in the brain, reduced iron availability, which affects myelination and neurotransmitter metabolism. Conversely, iron dyshomeostasis also contributes to neurodegenerative pathology. In this review, we synthesize recent evidence of how stress reprograms brain iron distribution and regulation, and we outline the mechanistic links between stress-induced iron dysregulation and neurological pathology. We also discuss the therapeutic implications (such as iron-chelation strategies) and highlight the three-way interplay among stress, iron metabolism, and neurodegeneration. These insights suggest that managing iron homeostasis may offer new therapeutic avenues for stress-related neural disorders.

## Simple Summary

Iron is an essential trace element whose homeostasis is critical for normal nervous system function. Increasing evidence indicates that stress perturbs iron metabolism and thereby contributes to the onset and progression of a spectrum of neurological diseases. Here, we synthesize the current knowledge of how iron levels change under stress, how those alterations reshape brain function, and the underlying molecular mechanisms. We focus on abnormal cerebral iron accumulation during stress and routes for iron entry across the blood–brain barrier. We further discuss the therapeutic potential of iron chelators and explore the three-way relationship among stress, iron metabolism, and neurological disease.

## 1. Introduction

**Figure 1:**
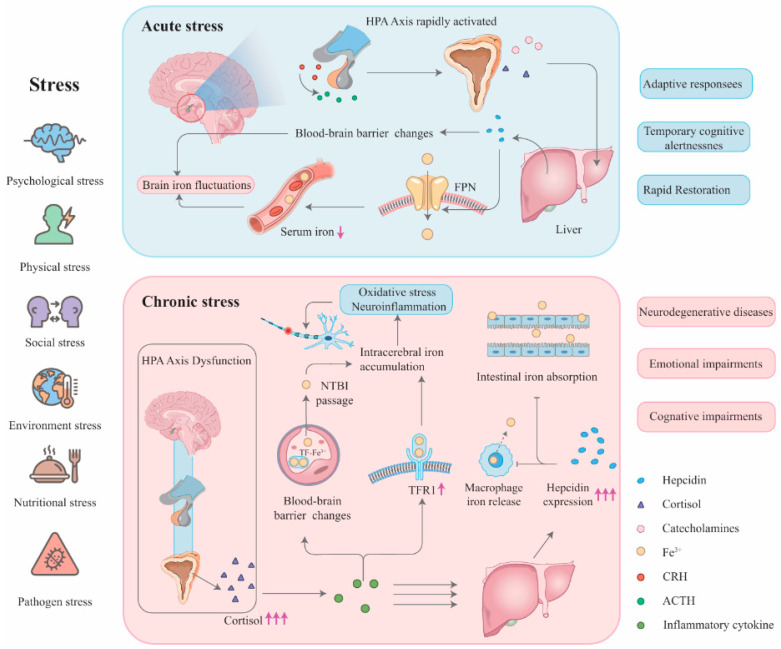
Mechanisms by which acute and chronic stress lead to different neurological outcomes through differential regulation of brain iron metabolism. Acute stress (**upper**): Brief activation of the HPA axis rapidly increases hepatic hepcidin levels, leading to systemic iron the sequestration via degradation of FPN and a subsequent decrease in serum iron. This process induces reversible cognitive adaptations without iron accumulation. Chronic Stress (**lower**): Sustained HPA axis activation and inflammation result in persistently elevated hepcidin levels, causing systemic “functional iron deficiency.” Concurrently, BBB integrity is compromised. On one hand, the brain may compensatorily upregulate TfR1 on the BBB to enhance iron uptake; on the other hand, BBB impairment may permit the abnormal leakage of NTBI. This leads to harmful iron accumulation in vulnerable brain regions, where iron-induced oxidative stress and neuroinflammation contribute to neuronal damage and increase the risk of neurodegenerative diseases and cognitive impairment. "↓"= a decrease in level; "↑"= an increase in level; "↑↑↑"= a substantial increase in level.

Stress has profound effects on physiology, and emerging evidence implicates stress in disrupting brain iron homeostasis. Iron is essential for normal neural function, and even slight imbalances in iron distribution or accumulation can contribute to neurological pathology. This review addresses the intersection of stress and brain iron metabolism (Figure 1), a topic of growing interest, given its links to neurodegenerative and neuropsychiatric disorders. We first outline how brain iron is regulated under normal conditions (Section 2) and then discuss how various forms of stress (particularly chronic stress) perturb iron transport and storage in the brain (Section 3). Next, we explore how these stress-induced iron dysregulations relate to specific neurological disorders (Section 4). Finally, we consider therapeutic approaches (especially iron chelators) and highlight open questions and future research directions (Section 5). By providing this overview, we aim to synthesize current knowledge and identify critical gaps in understanding the three-way relationship between stress, iron metabolism, and neurological disease.

## 2. Brain Iron Metabolism Under Physiological Conditions

### 2.1. Iron Transport Within the Central Nervous System

Successful iron transport requires the coordinated action of various proteins. Key proteins involved in iron transport include the following: Divalent Metal Transporter 1 (DMT1), which mediates intestinal iron absorption and iron transport through acidified endosomes [1,2]; Ferroportin (FPN), the only known membrane iron export protein [3]; Transferrin (Tf), the primary β-globulin in plasma that binds and transports iron [4]; Transferrin Receptor 1 (TfR1), which interacts with Tf to promote iron absorption [5]; Ferritin, which is an intracellular iron storage protein [6]; and hepcidin, which is a circulating peptide hormone that regulates iron homeostasis by mediating FPN degradation [7].

Iron is transported to the brain primarily in two forms: the iron–transferrin complex (TF-Fe^3+^) and non-transferrin-bound iron (NTBI). Mechanisms regulating iron uptake in the brain are highly stringent. For clarity, we address the transferrin-dependent iron uptake mechanism first, then later discuss how NTBI enters and affects the brain. The blood–brain barrier (BBB) is made up of endothelial cells, astrocyte, pericytes, and the basement membrane. The BBB is the primary barrier regulating iron entry into the brain parenchyma, collaborating with the choroid plexus (the blood–CSF barrier) to control the influx of iron and other substances into the central nervous system [8]. Endothelial cells of the BBB tightly restrict transcellular movement of iron, while the choroid plexus epithelium actively transports iron into the cerebrospinal fluid, together maintaining brain iron balance [9]. Brain microvascular endothelial cells (BMVECs) constitute the primary component of the BBB and serve as the target cells recognized by the TF-Fe^3+^ complex [10].

**Figure 2:**
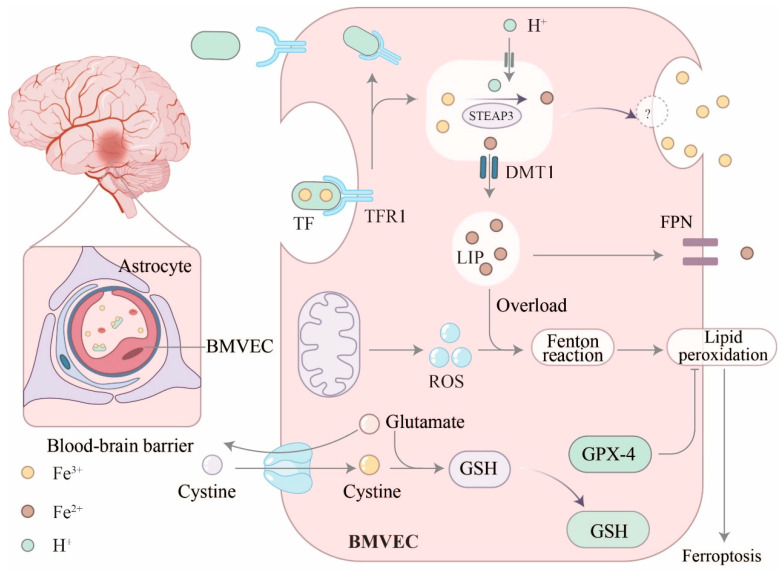
Iron-ion-targeted transport across the blood–brain barrier into the brain via BMVECs. Iron ions are transported to the brain in two forms: iron–transferrin complexes (TF-Fe^3+^) and non-transferrin-bound iron (NTBI). Iron is transported into labile iron pool (LIP). When LIP becomes iron-overloaded, it triggers the Fenton reaction, leading to iron-induced neuronal cell death. Beyond iron overload, excessive intracellular ROS levels and inactivation of the antioxidant enzyme GPX4 also cause intracellular peroxide accumulation, triggering iron-induced cell death. ? = unknown pathway or mechanism.

The uptake and circulation of the TF-Fe^3+^ complex involve several steps (Figure 2): (i) Due to tight junctions (TJs) between BMVECs, the TF-Fe^3+^ complex can only enter cells via a transcellular pathway (endocytosis) by binding to TfR1 on the BMVEC membrane, a process dependent on clathrin. (ii) The internalized Tf/TfR1 complex is membrane-enveloped to form vesicles, which are transported to and fuse with endosomes. (iii) Within the endoplasmic reticulum lumen of BMVECs, Fe^3+^ is released from Tf under acidic pH conditions (approximately 5.5), achieved by proton pump activity. (iv) Fe^3+^ is reduced to Fe^2+^ with the participation of iron reductases such as STEAP3. (v) Fe^2+^ then crosses the endoplasmic reticulum membrane via DMT1 into the labile iron pool (LIP) in the cytoplasm. (vi) Non-iron-bound Tf (apo-Tf) carrying TfR1 is returned to the membrane, where apo-Tf is released into the bloodstream [11,12,13,14].

Nearly all the blood vessels in the brain are surrounded by astrocyte: specialized cell units that maintain the brain’s ion and osmotic balance. When iron is released from BMVECS, it may be absorbed by the terminal astrocyte. As the most abundant neuroglia in the brain, the main role of astrocyte is to transport iron ions to other neuroglia and neurons, rather than storing them [15]. Fe^2+^ released from cells is rapidly oxidized to Fe^3+^ by ferroxidases (e.g., astrocyte-derived ceruloplasmin or neuronal hephaestin), enabling the iron to bind to transferrin in the interstitial fluid. The newly loaded transferrin can then deliver iron to TfR1-expressing cells throughout the CNS.

NTBI may have toxic effects in plasma and be almost undetectable under physiological conditions, but it may still be present. A hemoglobin molecule can bind two iron atoms, and, in general, about 70% of the iron-binding sites in the plasma transferrin pool are left unoccupied at any one time; this implies a transferrin saturation of about 30%, providing a considerable buffer against the generation of NTBI. When iron is overloaded, transferrin is oversaturated, leading to NTBI complexity. Due to its high redox activity, NTBI can react with biomolecules such as sugars, lipids, and proteins, and cause tissue peroxidation damage by producing harmful reactive oxygen species (ROS) in cells. Transport of NTBI relies on DMT1 and ferroreductases in traditional models. Fe^3+^ is reduced to Fe^2+^ by ferroreductase and then transported across the cell membrane with H^+^ through DMT1. Furthermore, DMT1-mediated NTBI transport is pH-dependent [16,17].

An increase in local H^+^ in the brain promotes the transport of NTBI, leading to Fe^2+^ accumulation in the nervous system. In fact, even under physiological conditions, astrocyte, oligodendrocyte, and microglia ingest iron in proportion through the NTBI because they do not express enough TF to meet their physiological needs [18]. However, when iron is overloaded, the uptake of NTBI increases accordingly, which may exacerbate intracellular oxidative stress. It has been found that Ferritin Heavy Chain (FTH), as a ferritin, helps NTBI cross the BBB by binding to type 1 receptor 1 containing t-cell immunoglobulin and mucin domains in human endothelial cells [19]. Moreover, prion protein is a ubiquitously expressed plasma membrane glycoprotein that is most abundant in nerve cells and is able to facilitate NTBI transport through DMT1 [20]. These considerations for normal iron handling set the stage for understanding how stress can disturb iron balance. In the next section, we examine how stress-induced blood–brain barrier changes alter iron transport.

### 2.2. Iron Uptake by Neural Cells

Under normal conditions, all iron entering the body originates from dietary sources. Duodenal epithelial cells transport dietary iron via DMT1 on their apical membrane [21]. After dietary iron enters intestinal cells through DMT1, it may bind to proteins and remain stably intracellular or be transported into systemic circulation via the sole iron transporter (located on the basolateral membrane). Beyond dietary iron absorption, iron recycled by macrophages represents another significant source. Macrophages absorb substantial iron by phagocytosing and degrading aged red blood cells and other senescent or damaged cells. Within macrophages, phagocytosed iron may be stored in ferritin after entering the cytoplasm, a process regulated by iron response element-binding protein (IREBP) [22]. Concurrently, iron can be transported into the extracellular fluid via ferroportin.

**Figure 3:**
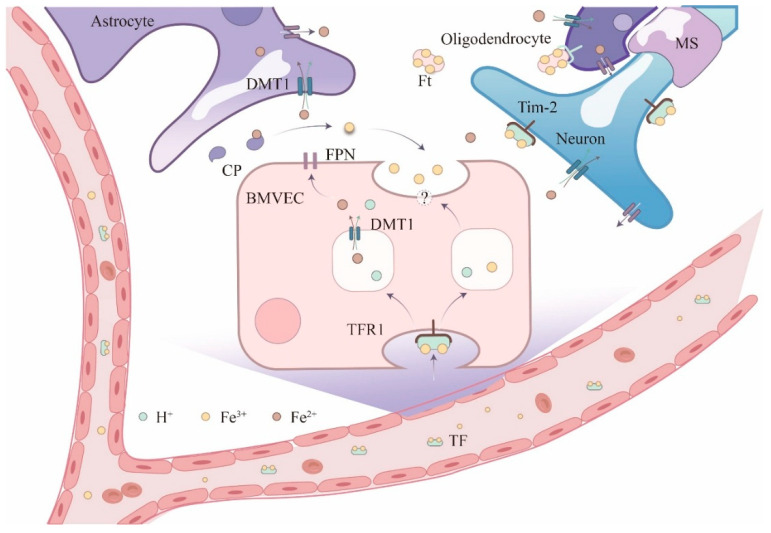
Brain iron metabolism. Astrocytes absorb iron from the circulatory system and distribute it to other cells within the central nervous system, facilitating intercellular iron transport. Oligodendrocytes may take up iron via the ferritin receptor Tim-2, via DMT1 for non-ferritin-bound iron, or via other non-vesicular iron ion uptake mechanisms. Neurons and microglia can take up iron via the transferrin–transferrin-receptor pathway and release iron through ferritin transporters. Neuronal axons are enveloped by myelin sheaths, a structure synthesized by oligodendrocytes in an iron-dependent manner. Solid arrows indicate the direction of iron flow. TFR1 = Transferrin receptor 1. TF = Transferrin. Fe^2+^ = Ferrous ion. Fe^3+^ = Ferric ion. BMVEC = Brain microvascular endothelial cells. CP = ceruloplasmin. FPN = ferritin transport protein. DMT1 = divalent metal transporter 1. Ft = ferritin. MS = myelin sheath. ? = unknown pathway or mechanism.

Different brain cells absorb iron through distinct mechanisms (Figure 3). Transferrin is synthesized in the brain by the choroid plexus and oligodendrocytes, but is secreted exclusively by the choroid plexus. Researchers suggest that neurons acquire most iron through the transferrin–transferrin-receptor system and likely export iron via FPN, as many neurons co-express Tf and FPN [23]. The astrocyte do not express TfR, but may absorb iron at the BBB through DMT1, which is expressed in foot processes associated with the BBB [24]. CP, a protein expressed by astrocyte in the CNS, indirectly regulates iron uptake and metabolism in nerve cells because the astrocyte foot wraps around the capillary; ceruloplasmin on its surface can oxidize Fe^2+^ to Fe^3+^, promoting the binding of iron to transferrin, thus affecting the subsequent receptor-mediated uptake process, while CP deficiency may lead to Fe^2+^ accumulation and oxidative damage, with indirect effects on iron availability in nerve cells [25]. In addition to astrocytes, other glial cells such as oligodendrocytes play critical roles in brain iron management, particularly as the iron-rich myelinating cells of the CNS.

As important as astrocytes are in regulating iron export and redistribution, oligodendrocytes also play a crucial but distinct role in brain iron management, particularly in relation to myelination. Oligodendrocytes harbor substantial iron. These may extract iron from adjacent blood vessels or uptake ferritin from the interstitium via ferritin receptors. Within oligodendrocytes, iron primarily exists as ferritin and transferrin [26]. Microglia have been reported to express both TfR and FPN [27]. While oligodendrocytes are the primary iron-containing cells in the adult brain, during brain development, microglia are the earliest cell type to accumulate iron (in the form of ferritin deposits), with iron deposition subsequently shifting to oligodendrocytes [28].

Neuronal iron uptake is primarily mediated by the transferrin receptor pathway, which relies on the Fe^3+^ valence state and the cooperation of plasma transferrin. DMT1 represents another crucial iron transporter. Its iron transport activity depends on an acidic environment, and DMT1 exhibits significant expression in neurons and brain endothelial cells, with its function closely linked to iron uptake [29]. Recently, H-ferritin has been demonstrated to supply iron to oligodendrocytes in both rodents [30] and humans [31]. In summary, each neural cell type manages iron differently; we consider below how stress hormones and inflammation perturb these finely tuned processes.

### 2.3. Intracellular Iron Storage and Function

Iron is an oxidizing metal whose homeostasis is tightly regulated in healthy cells. Most iron exists in the form of binding to proteins, such as the heme compound hemoglobin, in red blood cells; iron is also found in storage proteins (such as Ferritin, Hemosiderin, and Myoglobin). Cells do not contain free iron, but in the cytoplasm, a small amount of iron is found bound in a loose form. This hard-to-define collection of ferrous ions is known as the “Labile Iron Pool” [32]. This loosely bound and metabolically active ferrous iron can be transported to different cells to meet various metabolic demands. Iron from the LIP can be used by mitochondria to synthesize a heme and iron–sulfur cluster, and it can also synthesize ferritin in the cytoplasm or be oxidized and stored in ferritin [33]. Under normal conditions, cells maintain normal function by providing adequate iron while limiting iron toxicity by controlling iron metabolism. The LIP in these cells accounted for only about 5% of the total intracellular iron content [32].

Ferritin is a large, globular protein of 24 subunits that can accommodate up to 4500 iron molecules [34], making it essential for maintaining iron balance in cells. The main function of ferritin is to regulate intracellular iron concentration, prevent the accumulation of free iron, and reduce the oxidative stress and cell damage caused by iron excess. Ferritin can also be secreted into the plasma, and the concentration of ferritin in plasma is closely related to intracellular iron storage, so it is a good indicator for assessing iron storage [35]. When ferritin is released, iron can be efficiently removed from ferritin and released from the cell via the iron transporter, which also acts as a “Safety valve” to prevent an iron overload in the cells.

**Figure 4:**
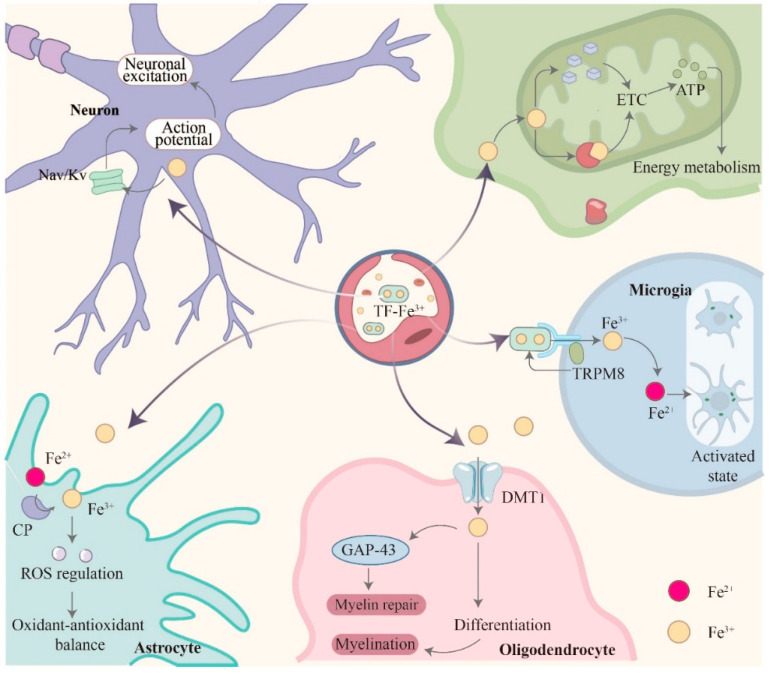
Utilization of iron in the brain following transport across the blood–brain barrier under physiological conditions. The image above depicts iron entering the brain via BMVECs across the blood–brain barrier (BBB) under normal physiological conditions. Once inside, iron is utilized in multiple physiological processes, including maintaining cellular energy metabolism and mitochondrial function, regulating neuroinflammation and immune surveillance, supporting neural regeneration and repair mechanisms, antioxidant defense and redox balance, and modulating neural signaling pathways.

Once in the brain, iron participates in a variety of physiological processes to maintain the proper functioning of the central nervous system (Figure 4). Iron (mainly Fe^2+^/Fe^3+^), as an essential cofactor of mitochondrial electron transport chain complex, is directly involved in the process of ATP synthesis. Recent studies have shown that iron homeostasis in mitochondria is essential for the maintenance of neuronal energy metabolism. Iron supports cellular oxidative phosphorylation processes by participating in the synthesis of heme and iron-sulfur clusters [36]. Regulation of neuroinflammation and immune surveillance: As central nervous system immune cells, microglia iron metabolism directly regulates neuroinflammation. Under physiological conditions, iron enters the microglia through the transferrin receptor-mediated uptake pathway and participates in the maintenance of immune homeostasis. TRPM8 ion channels have been shown to regulate transferrin-mediated Fe^3+^ uptake and affect the activation state of microglia [36]. Supporting mechanisms of nerve regeneration and repair: Studies of iron-synergistic biomaterials have shown that iron-based biomaterials can be used to enhance the ability of nerve regeneration and repair; Fe^3+^ can promote axonal regeneration and myelin sheath repair through the coordination of metal ions. Under physiological conditions, iron is involved in oligodendrocyte differentiation and myelination, which are critical for maintaining nerve conduction velocity, antioxidant defense, and redox balance [37]. Iron excess may induce oxidative stress, but it participates in antioxidant defense through ceruloplasmin-mediated Fe^2+^/Fe^3+^ conversion at physiological concentrations. Ceruloplasmin is expressed in astrocyte, and its oxidase activity prevents Fe^2+^ accumulation and maintains redox homeostasis in the brain [25]. Regulation of neural signal transmission: Iron affects neuronal excitability by regulating voltage-gated ion channel activity. Studies have found that Fe^3+^ can regulate the action potential of dorsal root ganglion neurons, which is of great significance to maintaining normal nerve conduction in the physiological range [27].

These results suggest that brain iron maintains the normal function of the central nervous system by participating in energy metabolism, immune regulation, nerve regeneration, antioxidant defense, and signal transduction. The realization of these functions depends on the strict regulation of iron homeostasis, and any imbalance may lead to neuropathological changes.

### 2.4. Iron Regulation and Export

The major hormone regulating iron homeostasis is Hepcidin, a liver hormone [27]. Hepcidin was initially found to have antibacterial properties and was later shown to control the release of iron from cells [38]. Hepcidin binds to the iron exporter ferroportin (FPN), leading to its internalization and degradation, thereby reducing intracellular iron excretion and lowering free iron concentrations in plasma. The major functions of Hepcidin include maintaining stable iron storage, providing adequate substrates for Erythropoiesis, and limiting microbial utilization of iron. Iron deficiency and Erythropoietin inhibit hepcidin production to support Erythropoiesis and other physiological needs [39], while iron overload and inflammation promote hepcidin production to protect cells from toxic effects [40]. Dysregulation of hepcidin is associated with multiple pathological conditions, with hepcidin deficiency leading to diseases such as inherited iron overload, while excess hepcidin is associated with iron deficiency anemia in chronic inflammatory diseases.

Iron homeostasis is mainly maintained by iron regulatory proteins (IRPs). IRPs can regulate the transcription of iron-related proteins by binding to the iron-responsive element (IRE) region in the untranslated region (UTR) of the mRNA [41]. When cellular iron concentrations are low, IRPs bind to the 5′ UTR of ferritin mRNA and the 3′ UTR of TfR1 mRNA, thereby blocking translation of the former and protecting the latter from degradation. Collectively, this mechanism maintains intracellular ferritin at low levels when iron storage is not required, while ensuring that more transferrin receptors are expressed on the cell surface, thus maximizing iron input. Conversely, when cellular iron levels are elevated, IRP does not bind IRE, thus allowing for ferritin translation and exposing TfR1 mRNA for degradation [42], which prevents iron uptake and supports iron storage. The main purpose of the IRP-IRE regulatory system is to ensure that cells are neither iron deficient nor supersaturated. This is by far the best-studied mechanism, but it is not the only one. Other factors such as hypoxia, hormones, and cytokines also have regulatory effects on iron-related genes [43].

Unlike other metals, iron leaves the body in a very limited way. The only normal way for iron to leave the body is through the shedding of epithelial cells (ferritin) in the small intestine. Therefore, the absorption of dietary iron in the small intestine must be tightly regulated to ensure adequate absorption to meet the body’s demand for iron, while preventing oxidative damage to cells and tissues due to excessive iron content [44].

## 3. Effect of Stress on Brain Iron Metabolism

### 3.1. Stress and Brain Iron Transport

Stress can disrupt the integrity of the blood–brain barrier (BBB), which normally maintains the stable environment of the central nervous system. For example, in mice exposed to chronic social defeat stress (CSDS), chronic stress was found to disrupt BBB integrity. Notably, female mice exposed to CSDS showed a significant downregulation of the tight junction protein claudin-5 in the prefrontal cortex (PFC) [45,46,47], indicating a compromised BBB. As the BBB’s integrity is reduced, iron and other substances can more easily penetrate into brain tissue, potentially contributing to neuroinflammation and damage [48,49]. In addition, the tight junction protein ZO-1 was found to be significantly reduced in iron-overloaded mice, suggesting that reduced BBB integrity, leading to iron leakage into the brain, may play a role in the pathogenesis of iron overload, persistent iron overload may lead to chronic neuroinflammation, neurodegeneration, and cognitive decline [50,51]. In summary, stress-induced BBB impairment allows for excess iron to infiltrate the brain, which can drive oxidative injury to neurons and glia. This iron-mediated neural damage provides a direct link between BBB disruption and neurological disorders under chronic stress.

Under chronic stress, the permeability of the blood–brain barrier changes significantly, which is closely related to many neurological disorders. Studies have shown that the release of inflammatory mediators (such as cytokines) causes dysfunction of the BBB endothelial cells, resulting in reduced expression of tight junction proteins and thus increased BBB permeability [52,53]. For example, cytokines such as IL-6 can promote the transmembrane transport of iron by regulating iron transporters and at the same time aggravate oxidative stress, forming a vicious cycle. In addition, the inflammatory reaction in the brain also leads to the dysregulation of DMT1 and TfR, which further leads to the accumulation of iron in the brain [54,55]. Therefore, the BBB is the key barrier for iron to enter the brain. Stress will change the permeability of the BBB by releasing inflammatory mediators and other substances, which will affect the transport of iron and ultimately lead to iron overload and neuronal damage in the brain.

### 3.2. Stress and Cellular Iron Uptake

#### 3.2.1. Stress and Iron Uptake in Neural Cells

Different brain cell types acquire and handle iron via distinct mechanisms, and chronic stress can perturb these pathways in a cell-type-specific manner (Table 1). Neurons primarily uptake iron through TfR1-mediated endocytosis of transferrin-bound iron [56,57], and many neurons also express FPN to export excess iron [58]. Under stress, elevated glucocorticoids and catecholamines directly influence neuronal iron homeostasis—for example, corticosterone exposure upregulates neuronal TfR1 and DMT1 expression while downregulating FPN, driving greater iron influx and retention in neurons. In contrast, astrocytes and microglia utilize partly distinct iron uptake routes and serve supportive roles in brain iron distribution. Astrocytes do not significantly express TfR1, but can take up non–transferrin-bound iron via DMT1 at perivascular endfeet [59]. They also secrete ceruloplasmin to oxidize Fe^2+^ to Fe^3+^, facilitating transferrin loading and neuronal uptake. Under inflammatory stress, astrocytes become reactive and may increase the synthesis of hepcidin [60], which acts locally to further trap iron in cells by degrading ferroportin. Microglia, the brain’s resident macrophages, express both TfR1 and ferroportin and can avidly phagocytose iron-rich debris [61]. During chronic psychological stress, microglia often shift to a pro-inflammatory state (M1), sequestering iron in response to elevated hepcidin and downregulating ferroportin [62,63]. This leads to microglia iron loading, as observed in models of social defeat stress where activated microglia accumulate ferritin-bound iron and produce cytokines that influence neuronal iron uptake. Meanwhile, iron’s availability to oligodendrocytes (which require abundant iron for myelination) can become restricted if astrocyte and microglia iron metabolism function is disturbed. Indeed, microglia and oligodendrocytes normally maintain a dynamic partnership in iron cycling, and iron imbalance in microglia can impair oligodendroglia iron supply and myelin integrity [64]. In summary, stress hormones and inflammatory signals tend to enhance neuronal iron uptake (through upregulated TfR1/DMT1) while promoting iron sequestration in glia (via hepcidin–ferroportin axis dysfunction) [65]. This differential effect upsets the normal neuron–glia iron exchange: neurons may experience toxic iron accumulation or, in other contexts, iron insufficiency for neurotransmitter synthesis, whereas microglia become iron-overloaded and pro-oxidative [66]. Such cell-specific iron dysregulation under chronic stress is a key factor linking stress to neural injury and degeneration.

#### 3.2.2. Glucocorticoids and Catecholamines Directly Tune Neuronal Uptake

Acute restraint stress activates the sympathetic system, elevating norepinephrine (NE) from adrenal medulla and locus coeruleus. β2-adrenergic signaling (cAMP/PKA/CREB) increases TfR transcription and membrane localization, enhancing Tf-Fe^3+^ endocytosis and raising intracellular iron. In chronic unpredictable mild stress (CUMS), corticosterone acting on neuronal glucocorticoid receptors (GR) upregulates DMT1 (promoting Fe^2+^ influx) while reducing FPN translation and membrane presence (curtailing efflux)—a “more in, less out” program that drives iron accumulation [67].

#### 3.2.3. Neuroinflammation Drives Microglia Iron Overload

Stress polarizes microglia toward an M1-like pro-inflammatory state, releasing IL-6 and TNF-α that activate neuronal JAK–STAT3 and NF-κB, upregulating TfR and DMT1 and fueling an inflammation–iron-overload feedback loop [68]. TNF-α further elevates nuclear receptor coactivator 4 (NCOA4)-mediated ferritinophagy, liberating stored iron into the cytosolic LIP and amplifying iron-dependent oxidative injury [69].

#### 3.2.4. Oxidative Stress: ROS and Iron Metabolic Reprogramming

Excess superoxide under stress converts IRP1 from its Fe–S enzymatic form to an RNA-binding form that represses ferritin translation and stabilizes TfR mRNA, tipping the balance toward uptake over storage [25]. Mitochondrial ROS in chronic stress can inhibit (ATP-Binding Cassette Transporter B8) ABCB8-dependent mitochondrial iron export, causing matrix iron buildup and Fenton chemistry that damages mtDNA and respiratory complexes—a self-propagating loop [36].

Chronic-stress-induced oxidative burden not only alters IRP activity, but also activates intracellular iron mobilization pathways. Excess ROS can damage iron–sulfur clusters in IRP1, converting it to an RNA-binding form that simultaneously increases iron uptake and reduces iron storage: IRP1 binds to IREs on ferritin mRNA to block ferritin translation, while stabilizing TfR1 mRNA to enhance its expression [25]. Moreover, NCOA4-mediated ferritinophagy is stimulated during stress, leading to excessive iron release from ferritin stores. NCOA4 acts as a selective autophagy receptor for ferritin, targeting it to autophagosomes for lysosomal degradation [69]. Under chronic stress or elevated catecholamine signaling, ferritinophagy can be exacerbated—for instance, β-adrenergic/cAMP signaling has been shown to increase ferritin turnover, likely via NCOA4, causing a surge of free iron in the cytosol [70]. The combined effect of heightened IRP1 activity (suppressing iron sequestration in ferritin) and upregulated ferritinophagy is a vicious cycle of iron unloading: previously safely stored iron is liberated into the cytosolic pool, where it participates in Fenton chemistry to generate hydroxyl radicals. Indeed, chronic stress elevates mitochondrial ROS, and if ABCB8-mediated mitochondrial iron export is concurrently impaired, iron accumulates in mitochondria and amplifies oxidative injury to mitochondrial DNA and enzymes. In parallel, IRP2 (which is normally degraded when iron is plentiful) may escape degradation under fluctuating redox conditions, further skewing the expression of iron metabolism genes [71,72]. Collectively, these post-transcriptional and post-translational dysregulations—IRP/IRE imbalance and ferritinophagy hyperactivation—markedly increase the intracellular free iron level. The consequence is pervasive oxidative damage to lipids, proteins, and nucleic acids via iron-catalyzed formation. This mechanism links cellular stress to iron-driven neurotoxicity and sets the stage for iron-dependent cell death processes in the brain.

### 3.3. Stress and Iron Storage/Utilization

In chronically stressed rats, both total and non-protein-bound iron levels were found to rise in several brain regions (frontal cortex, hippocampus, striatum), accompanied by increased TfR1 expression and histological iron deposition [73,74]. This preclinical finding supports the idea that prolonged stress can drive brain iron accumulation and redistribution [75]. Inflammation-induced ferritinophagy (via TNF-α → NF-κB → NCOA4) liberates stored iron, expanding the LIP [67]. Elevated LIP deranges energy metabolism (mitochondrial Fenton chemistry, ETC damage), reshapes epigenetics (Fe^2+^ cofactor for dioxygenases), and perturbs neurotransmitter synthesis (e.g., by modulating tyrosine hydroxylase) [25,36].

### 3.4. Stress and Regulation of Brain Iron Homeostasis

Chronic stress thus produces a dynamic imbalance in the hepcidin–ferroportin–TfR–DMT1 regulatory network that governs systemic and brain iron homeostasis. Activation of the HPA axis persistently elevates hepcidin, which internalizes ferroportin and traps iron in storage sites (liver, spleen, and microglia) at the expense of circulating iron [76]. This leads to an apparent “functional iron deficiency” despite ample iron stores—a phenomenon reflected by reduced brain iron uptake in some chronic stress models. Conversely, stress-induced sympathetic outflow and inflammatory signaling drive increased iron import and intracellular retention in neural tissues. Norepinephrine via β-2-adrenergic receptors boosts neuronal and microglia TfR1 expression while concurrently reducing ferroportin availability on cell membranes [77]. The result is that brain cells (especially neurons and activated microglia) continue to import iron, but cannot effectively export or safely store it.

Over time, this feed-forward dysregulation breaks the equilibrium between iron uptake, utilization, and release. Elevated hepcidin and diminished ferroportin cause iron to accumulate in brain macrophages (microglia) and within the BBB endothelium, whereas neurons experiencing high catecholaminergic and oxidative stress upregulate DMT1/TfR1 and import more iron than they can utilize or export [65]. Such chronic perturbation of the iron flux network leads to region-specific brain iron accumulation alongside peripheral iron restriction. For example, prolonged stress in rodents triggers iron build-up in the limbic system and basal ganglia even as serum iron falls, implicating a redistribution driven by the hepcidin–ferroportin axis. Likewise, sustained IRP activation (due to ongoing ROS) tilts cells toward iron influx over storage, compounding the imbalance. This dynamic misregulation of iron homeostasis under chronic stress is believed to underlie neuropathological changes in stress-related disorders. Notably, brain regions of depressed patients show abnormal iron deposition and altered iron-handling protein expression, consistent with a prolonged stress state disrupting iron equilibrium [78]. Restoring balance to the hepcidin–ferroportin–TfR–DMT1 network may therefore be a critical therapeutic strategy to mitigate neural damage from chronic stress.

#### 3.4.1. HPA Axis—Glucocorticoids

HPA axis activation during stress elevates glucocorticoids (cortisol/corticosterone), which can upregulate hepcidin and alter neuronal iron handling; corticosterone disrupts iron metabolism in hippocampal neurons in vitro [79]. In adolescents, higher self-reported life-event stress is associated with increased IL-6 and hepcidin [80]. Sustained glucocorticoid exposure in chronic stress thus limits intestinal absorption and favors storage, while stress-inflammation further elevates hepcidin, compounding functional iron deficiency and maldistribution.

#### 3.4.2. Sympathetic Axis—Norepinephrine

Acute/chronic NE signaling elevates TfR and reduces FPN membrane localization via β-adrenergic cAMP pathways, increases endosomal acidification and Fe^3+^ → Fe^2+^ release (facilitated by TRPM8-dependent microenvironmental changes), promotes ferritinophagy (via NF-κB/NCOA4), and disrupts IRP–IRE set-points—collectively expanding the LIP, triggering Fenton chemistry, and impairing mitochondrial function [25,36,67]. These events form a mechanistic bridge from stress to iron-mediated neurotoxicity and psychiatric symptomatology, and suggest iron-targeted interventions (e.g., chelators, anti-TfR strategies).

### 3.5. Ferroptosis in Stress-Related Neuropathology

Ferroptosis is an iron-dependent non-apoptotic cell death pathway characterized by overwhelming lipid peroxidation and reactive oxygen species generation [81]. In ferroptotic cells, excessive free iron catalyzes the formation of hydroxyl radicals and peroxides that peroxidize polyunsaturated fatty acids in membranes, while antioxidant defenses (especially glutathione (GSH) and GPX4 activity) are impaired [82]. Emerging evidence implicates ferroptosis as a key link between chronic stress and neurodegeneration. Under psychological stress, the cumulative iron dyshomeostasis described above—elevated labile iron, glutathione depletion, and persistent oxidative stress—creates conditions ripe for ferroptotic cell death in the central nervous system. For instance, in mouse models of chronic unpredictable stress, neurons in the hippocampus and prefrontal cortex exhibit molecular hallmarks of ferroptosis, including lipid ROS accumulation and downregulation of SLC7A11/GPX4, alongside behavioral deficits [83]. In these models, pharmacological inhibition of ferroptosis (using lipophilic antioxidants or iron chelators) reverses the neuronal damage and ameliorates depression-like behaviors, highlighting ferroptosis as a driver of stress-induced neuropathology [84,85].

Similarly, inflammatory stressors that often accompany psychological stress can precipitate ferroptosis. In a recent study, systemic lipopolysaccharide (LPS) exposure (mimicking chronic inflammation) led to excess brain iron loading and ferroptotic injury in mice, which was attenuated by the iron-chelator deferoxamine [86]. Post-mortem and neuroimaging analyses of patients with major depression and chronic stress exposure have also revealed increased brain iron in specific regions along with evidence of lipid peroxidation, consistent with ferroptotic processes. Mechanistically, chronic stress elevates corticosterone and glutamate, which can deplete astrocytic glutathione and diminish neuronal antioxidant capacity, lowering the threshold for ferroptosis [87]. Excess iron in stress-vulnerable brain regions (e.g., hippocampus, basal ganglia) exacerbates peroxidative damage to neurons, while microglia activation supplies additional ROS, together tipping the balance toward ferroptotic cell death. The ferroptotic loss of neurons and glia is increasingly recognized as an important contributor to stress-related neurodegenerative changes, including dendritic atrophy, demyelination, and synaptic dysfunction. Encouragingly, targeting ferroptosis holds therapeutic promise: in models of chronic stress and neuroinflammation, agents that reduce iron-catalyzed lipid peroxidation (such as ferrostatins, liproxstatins, or novel nanocluster inhibitors) confer neuroprotection and improve cognitive outcomes [88,89,90].

In summary, ferroptosis represents a pathological end-point of prolonged stress-induced iron misregulation and oxidative injury. By introducing an iron-dependent cell death mechanism, stress not only perturbs neurotransmission and plasticity, but can also irrevocably damage neural cells. Ongoing research in both animals and humans is unraveling this lethal connection, positioning ferroptosis as a central mediator whereby chronic stress translates into neurodegeneration and a potential target to break the link between psychological stress and brain disease.

## 4. Brain Iron Metabolism Abnormalities in Organisms

The brain is the second most iron-rich organ after the liver. Iron is widely involved in fundamental biological processes in brain cells, including oxygen transport, DNA synthesis, antioxidant enzyme activity, mitochondrial respiration, myelin synthesis, and neurotransmitter synthesis and metabolism [91]. Iron homeostasis imbalance can induce free radical damage, inflammation, protein misfolding or aggregation, mitochondrial dysfunction, and neurological dysfunction [92]. An excessive accumulation of iron in specific regions of the brain is also a pathological feature of neurodegeneration, and an imbalance in iron homeostasis can trigger Friedreich ataxia [93].

Intracellular excess iron, especially Fe^2+^, is a powerful pro-oxidant, which mainly catalyzes the Fenton reaction and damages mitochondria to produce a large amount of ROS, which in turn leads to lipid peroxidation, oxidative stress, and oxidative stress, which leads to cell death, which is a key process in ferroptosis [94,95]. In many cases, the relationship between iron overload and oxidative stress is bidirectional: iron accumulation promotes ROS generation, which in turn promotes further iron accumulation through multiple mechanisms [96,97]. By inhibiting ROS production in mitochondria, it can effectively alleviate the cell damage caused by iron overload. In addition, increased tissue iron content was observed in the spleen and liver of iron-overloaded and β-thalassaemia mice, accompanied by cell hyperplasia and hemosiderin deposition [50].

### 4.1. Iron and Neurodegenerative Diseases

Among neurodegenerative disorders, Parkinson’s and Alzheimer’s diseases have been strongly linked to brain iron misregulation.

AD (Alzheimer’s disease, AD) is a central nervous system degenerative disease. Recent studies have shown that iron metabolism disorder play an important role in the pathogenesis of AD. The expressions of FTL and FTH in the brain of AD patients increased significantly, while the expressions of DMT1 and FPN decreased. In addition, in AD, xCT expression, and lipid peroxidation product 4-HNE levels were significantly increased, while GSH levels were significantly decreased, suggesting that excess iron may impair the brain’s antioxidant capacity, leading to the occurrence of ferroptosis [26].

PD (Parkinson’s Disease, PD) is a neurodegeneration that primarily affects motor control and is characterized by the selective loss of dopaminergic neurons, particularly in the substantia nigra region. Studies have shown that abnormal deposition of intracranial iron is one of the important pathogenic mechanisms of PD, which is manifested by the gradual increase of iron content in the substantia nigra of PD patients [98]. Autopsies of PD patients showed significantly higher levels of total iron and ferric iron in the substantia nigra [99,100], and activation of the microglia was observed. In addition, PD patients’ microglia are rich in ferritin, suggesting that iron metabolism and storage are active in the microglia [101]. In PD patients and PD models triggered by 6-hydroxydopamine (6-OHDA) and 1-Methyl-4-phenyl-1,2,3,6-tetrahydropyridine (MPTP), DMT1, which is responsible for transporting ferrous iron, is upregulated in the substantia nigra, which in turn leads to the degeneration of dopaminergic neurons. In vitro studies have also confirmed that increasing the level of DMT1 promotes iron transport and exacerbates oxidative stress in cells [43]. Degradation of DMT1 by the ubiquitin–proteasome pathway during iron accumulation may be a key factor in DMT1 dysregulation [102].

Epileptic seizure (SE) is a chronic brain disease characterized by sudden and unexplained seizures caused by abnormal discharge of neurons in the brain, and the seizures of the disease are recurrent and transient [103,104,105]. In recent years, studies have shown that epilepsy is closely related to iron metabolism. Patients with SE, for example, have elevated iron levels in neurons and astrocyte and oxidative damage to their cells [106]. A subset of genes involved in iron metabolism showed lower expression of iron uptake (TfR), iron binding (FTH, FTL), and iron export inhibition (HAMP) compared to postmortem control tissues, suggesting that iron metabolism may be involved in the regulation of iron homeostasis; the genes (STEAP3, DMT1) and cellular iron export (FPN1, HEPH) involved in the transition and export of iron activity state in vivo were over-expressed [107]. In the rat model of electrically induced SE, ferritin was found to be strongly expressed in the activated microglia in the rat hippocampus when seizures occurred, indicating an accumulation of iron in the microglia. Studies have found that ferric iron overload does not induce epileptic activity, but leads to iron-dependent activity in neurons and upregulation of pro-inflammatory genes [106,108].

Huntington’s disease (HD) is a neurodegenerative disease that is inherited in an autosomal dominant manner caused by abnormal expansion of the CAG genetic code repeat in exon 1 of the Huntington gene (HTT) [109,110]. In quantitative susceptibility imaging of patients with pre-Huntington symptoms, significantly higher susceptibility values were found in the caudate, putamen, and globus pallidus, indicating increased iron content in these structures; however, magnetic susceptibility was significantly decreased in the substantia nigra and hippocampus, and negatively correlated with the structural volume in the caudate and putamen [111]. Elevated iron levels in striatal neurons, decreased expression of iron-reactive protein (IRP1/2) and TfR, and increased levels of iron transporter can be detected in HD mouse models, suggesting a role for iron in the pathogenesis of HD; this suggests the existence of an adaptive IRP response that reduces further iron uptake from elevated free iron levels. Intracerebroventricular injection of the iron chelator deferoxamine (DFO) ameliorated striatal pathology and motor performance in HD mice [112]. But a recent human HD brain imaging study found no correlation between MRI-measured brain atrophy and brain iron levels [113].

An imbalance in brain iron homeostasis is a contributing factor to the development of multiple neurological disorder. Abnormal iron deposition in specific brain regions was observed in AD, PD, SE, and HD. This imbalance in iron homeostasis leads to oxidative stress and mitochondrial structural damage in cells, which in turn leads to ferroptosis, which in turn promotes the development of neurodegeneration.

### 4.2. Iron and Neuropsychiatric Disorders

Recent studies have demonstrated significant associations between stress-related neuropsychiatric disorders and the dysregulation of the iron metabolism in the central nervous system. In patients with Major Depressive Disorder (MDD), multiple clinical studies using quantitative susceptibility mapping (QSM) have revealed significantly elevated iron concentrations in the basal ganglia regions, particularly in the globus pallidus and putamen [114,115]. Notably, this pattern of iron accumulation shows a positive correlation with disease severity, with abnormal iron deposition also observed in traditionally “iron-poor” regions such as the prefrontal cortex [116]. Furthermore, Post-Traumatic Stress Disorder (PTSD) rat models exhibit elevated iron concentrations in key brain regions such as the hippocampus, prefrontal cortex, and striatum [117].

Patients with Generalized Anxiety Disorder (GAD) have elevated serum iron levels [118]. Particularly noteworthy are case reports showing significant responses to iron chelator therapy in some patients with treatment-resistant anxiety disorders, suggesting that iron metabolism dysregulation may directly participate in the disease pathology [119].

Schizophrenia spectrum disorders similarly exhibit characteristic iron metabolism abnormalities. Meta-analyses show that patients with first-episode psychosis already demonstrate abnormal iron concentrations in the basal ganglia regions [120]. Brain iron assessment using quantitative susceptibility mapping in first-episode schizophrenia patients revealed significantly reduced QSM values in the bilateral substantia nigra, left red nucleus, and left thalamus, indicating decreased iron concentration in these gray matter nuclei [121,122]. A large-scale multicenter study found that serum ferroportin (FPN) levels are significantly elevated in patients with schizophrenia [123].

It is particularly noteworthy that these iron metabolism alterations in stress-related neuropsychiatric disorders may share common neurobiological mechanisms. Preclinical studies indicate that chronic stress can activate the hypothalamic–pituitary–adrenal (HPA) axis, leading to upregulated hepcidin expression and the consequent disruption of cerebral iron homeostasis [124]. Additionally, neuroinflammatory responses are considered an important bridge connecting stress, iron metabolism dysregulation, and neuropsychiatric symptoms [125,126].

These findings not only deepen our understanding of the pathological mechanisms of neuropsychiatric disorders, but also provide direction for developing new treatment strategies. Clinical application research on iron chelators and iron metabolism modulators in treatment-resistant depression is actively underway [127,128], suggesting that targeting iron metabolism may become a novel therapeutic approach for neuropsychiatric disorders.

## 5. Therapeutic Potential of Iron Chelators

Iron accumulation can directly damage neurons and glia through oxidative stress and by triggering ferroptosis [129]. Consistently, iron-chelation therapy has shown potential to slow neurodegenerative processes by reducing iron-induced toxicity [130,131]. For this reason, iron chelators are being explored as therapeutic agents in various neurological disorders. As an important drug, iron chelators are widely used to treat a variety of diseases caused by iron overload, such as certain types of cancer, neurodegeneration, and cardiovascular diseases. The commonly used iron chelators mainly include Deferoxamine (DFO), Deferiprone (DFP), and Deferasirox (DFX) [132,133]. DFO is a water-soluble chelating agent used to treat iron toxicity to prevent iron ion-induced cell damage by efficiently binding free iron ions. DFO is not highly bioavailable orally and is generally administered intramuscularly, subcutaneously, or intravenously. In addition, its half-life in plasma is very short, only 5–10 min [134]. DFP, the first oral iron chelator released in 1980, has higher bioavailability and tolerability, with a short half-life, and is suitable for long-term treatment. DFX is a synthetic oral iron chelator with a long half-life and 24 h chelation, so it can be used once a day. Unlike DFO, DFX can bind to Fe^3+^ and is highly lipophilic [135,136,137].

The role of an iron chelator is to enhance the solubility of iron, ameliorate the activity of iron, and reduce the toxicity of free iron to the body. Studies have shown that excessive accumulation of free iron could promote oxidative stress and damage cells, leading to a series of diseases. Iron chelators usually contain donor atoms of oxygen, nitrogen, or sulfur, which form coordination bonds with iron. Since the body’s ability to excrete excess iron is severely limited, chelation therapy reduces the toxic effects of iron overload on cells by combining non-Tf-bound iron to reduce the increase in free iron [138,139,140,141]. In many neurological disorders, abnormal iron deposits in the brain are thought to be a pathological feature. The abnormal accumulation of iron can not only directly lead to nerve cell damage, but also exacerbate the development of the disease by triggering inflammatory response and oxidative stress [142,143]. Iron chelators have been shown to slow the progression of neurodegeneration by clearing abnormal iron deposits from the brain. For example, DFO could ameliorate cognitive function in AD by reducing serum iron levels in the brain [142]. The use of DFO in a rat model of brainstem hemorrhage (BSH) could alleviate erythrocytolysis and iron deposition at the brainstem site, and can also reduce ROS levels and alleviate neuronal and myelin injury [144]. DFO is highlighted in many studies, as it is a long-established treatment for iron overload; however, newer chelators such as DFP and DFX are gaining attention in neurological research. Likewise, DFP has demonstrated neuroprotective effects in experimental models of Parkinson’s disease, given its ability to cross the BBB and reduce neuronal iron levels [145,146]. DFX, while effective for peripheral iron overload, is being evaluated for neurological conditions, but its efficacy may be limited by BBB permeability [147,148].

Iron chelators may also act on the body by ameliorating mitochondrial function. Studies have found that Kua, as a natural small-molecule compound, blocks the mitochondrial apoptosis pathway by directly sequestering cellular iron, inhibiting iron overload, scavenging 6-OHDA (a selective neurotoxin)-induced intracellular ROS, and reducing mitochondrial apoptotic pathway, reducing oxidative damage, and maintaining iron homeostasis [149]. Furthermore, in the LPS-induced mouse models of cognitive dysfunction and neuroinflammation, the use of DFO inhibited LPS-induced iron accumulation and modulated the imbalance in the expression of iron-metabolism-related proteins caused by LPS, suggesting that DFO may play a role in the pathogenesis of LPS-induced cognitive dysfunction and neuroinflammation [150]. Iron chelators alleviate iron overload-triggered cellular oxidative damage, mitochondrial dysfunction, as well as inflammatory responses at source by specifically integrating toxic free iron. Its neuroprotective effect in various neurological disorder models fully proves that regulating brain iron homeostasis is a promising therapeutic approach. Nonetheless, iron-chelation strategies face important limitations. DFO requires invasive administration and has a limited ability to cross the BBB, and its use can cause adverse effects (e.g., infusion site reactions, auditory and ocular toxicity at high doses) [151]. Deferiprone, while orally active and a BBB-penetrant, can induce agranulocytosis in a subset of patients, necessitating regular blood monitoring [152]. Deferasirox can cause hepatic and renal impairment [153]. Moreover, excessive iron removal could theoretically impair physiological iron-dependent processes. These side effects and pharmacokinetic limitations highlight the need for caution and further optimization of chelation therapy for brain disorders.

## 6. Conclusions

Chronic stress induces aberrant brain iron metabolism through the combined effects of elevated hepcidin (via HPA axis activation), increased oxidative stress, and BBB dysfunction. Together, these factors can cause functional iron deficiency in some neural cells and iron overload in others, ultimately contributing to neuronal injury and the development of neurological disorders. Our review underscores that iron misregulation is a key mediator linking stress to neural damage.

Notably, although the acute stress response may trigger a transient redistribution of iron, it is important to understand the mechanisms involved as adaptive mechanisms (e.g., iron storage in the body in the “Fight or flight” response to reduce oxidative damage or pathogen proliferation), but these changes are transient and reversible. Crucially, the transition to an adverse iron imbalance seems to depend on the duration and intensity of the stress. On the basis of current evidence, chronic stress patterns—often manifested as repeated or continuous exposures over weeks to months—are necessary to cause the ongoing pathophysiological changes described in this article. In contrast, this chronic stress continues to cause an imbalance in iron distribution, allowing for iron to accumulate in some cells (e.g., microglia) and become deficient in others (e.g., oligodendrocyte). This condition can lead to toxicity and impair neurological function.

Accordingly, therapeutic strategies that normalize brain iron levels, such as iron-chelating treatments, may mitigate stress-related neuropathology. Indeed, we discussed the potential of iron chelators to alleviate neuroinflammation and neuro-degeneration in models of chronic stress, although their clinical translation will require careful consideration of safety and delivery. In parallel, improving diagnostic approaches for iron dysregulation in the brain will be crucial. Advanced imaging modalities, such as MRI-based quantitative susceptibility mapping, allow for in vivo visualization of brain iron deposits and are increasingly used to link iron burden with disease severity [23,24]. Molecular diagnostics, for instance, measuring iron-handling proteins or hepcidin levels in cerebrospinal fluid or blood, could serve as surrogate indicators of central iron status. At the histological level, techniques like Prussian blue staining and electron microscopy have revealed iron accumulation within specific brain cells and organelles, providing direct confirmation of iron dyshomeostasis in affected regions. Developing non-invasive iron biomarkers and integrating these diagnostic tools into research and clinical practice will greatly aid in the early detection and monitoring of stress-related iron perturbations.

Notably, it remains unclear how acute stress responses differ from chronic stress in terms of iron redistribution—an important gap that future studies should address. Further investigation is also needed to determine how to safely target brain iron with therapeutics and to develop diagnostic tools (e.g., advanced imaging) for the early detection of iron dysregulation.

## Figures and Tables

**Table 1:** Iron metabolism characteristics of major CNS cell types and effects of stress.

Cell Type	Key Iron Uptake/Storage/Export Mechanisms	Changes Under Stress (Chronic Stress or Inflammation)	References
Neurons	Uptake: TfR1 (Fe^3+^-Tf), DMT1 (NTBI) Export: Ferroportin (FPN)	DMT1 ↑ TFR1 ↑ FPN ↓ (due to glucocorticoid and catecholamine effects) → iron accumulation	[56,57,58]
Astrocytes	Uptake: Primarily NTBI via DMT1 at endfeet (no significant TfR1) Export: Minimal expression of FPN	Hepcidin ↑ (inflammatory stress) → traps iron in astrocytes; unclear export changes	[59,60]
Microglia	Uptake: Phagocytosis, TfR1 Export: FPN	Shift to M1 state → iron uptake ↑ (phagocytosis, possibly TfR1 ↑ via cytokines), FPN ↑ (hepcidin from astrocytes/microglia) → microglia iron overload	[61,62,63]
Oligodendrocytes	Uptake: Transferrin (TfR1), ferritin (via ferritin receptors) Export: Not well-established (some FPN reported)	Iron uptake likely limited if astroglia/microglia sequester iron; chronic stress may reduce iron availability, potentially contributing to myelin defects (inferred)	[64]

The symbol ‘↑’ denotes an increase in expression level, while ‘↓’ denotes a decrease.

## Data Availability

No new data were created or analyzed in this study. Data sharing is not applicable.

## Abbreviations

1-Methyl-4-phenyl-1,2,3,6-tetrahydropyridine (MPTP), 6-Hydroxydopamine (6-OHDA), ATP-Binding Cassette Transporter B8 (ABCB8), Blood–Brain Barrier (BBB), Brain Microvascular Endothelial Cells (BMVECs), Brainstem Hemorrhage (BSH), Central Nervous System (CNS), Ceruloplasmin (CP), Chronic Social Defeat Stress (CSDS), Chronic Unpredictable Mild Stress (CUMS), Deferasirox (DFX), Deferiprone (DFP), Deferoxamine (DFO), Divalent Metal Transporter 1 (DMT1), Epileptic seizure (SE), Ferritin Heavy Chain (FTH), Ferroportin (FPN), Glucocorticoid Receptors (GR), Glutathione (GSH), Hephaestin (HP), Huntingtin Gene (HTT), Huntington’s disease (HD), Hypothalamic–Pituitary–Adrenal (HPA), Iron Regulatory Proteins (IRPs), Iron Response Element (IRE), Iron Response Element Binding Protein (IREBP), Iron-Transferrin Complex (TF-Fe^3+^), Labile Iron Pool (LIP), Lipopolysaccharide (LPS), Major Depressive Disorder (MDD), Non-iron-bound Tf (apo-Tf), Non-Transferrin-Bound Iron (NTBI), Norepinephrine (NE), Parkinson’s Disease (PD), Prefrontal Cortex (PFC), Quantitative Susceptibility Mapping (QSM), Reactive Oxygen Species (ROS), Substantia Nigra (SN), Tight Junctions (TJs), Transferrin (Tf), Transferrin Receptor 1 (TfR1), Untranslated region (UTR).

## References

[1] Canonne-Hergaux F., Zhang A.S., Ponka P., Gros P. (2001). Characterization of the iron transporter DMT1 (NRAMP2/DCT1) in red blood cells of normal and anemic mk/mk mice. Blood.

[2] Jabado N., Canonne-Hergaux F., Gruenheid S., Picard V., Gros P. (2002). Iron transporter Nramp2/DMT-1 is associated with the membrane of phagosomes in macrophages and Sertoli cells. Blood.

[3] Babu K.R., Muckenthaler M.U. (2016). miR-20a regulates expression of the iron exporter ferroportin in lung cancer. J. Mol. Med..

[4] Ju Z.H., Li Q.L., Huang J.M., Hou M.H., Li R.L., Li J.B., Zhong J.F., Wang C.F. (2011). Three novel SNPs of the bovine Tf gene in Chinese native cattle and their associations with milk production traits. Genet. Mol. Res..

[5] Gao G., Li J., Zhang Y., Chang Y.Z. (2019). Cellular Iron Metabolism and Regulation. Adv. Exp. Med. Biol..

[6] Kotla N.K., Dutta P., Parimi S., Das N.K. (2022). The Role of Ferritin in Health and Disease: Recent Advances and Understandings. Metabolites.

[7] Ganz T. (2016). Hepcidin. [Rinsho Ketsueki] Jpn. J. Clin. Hematol..

[8] Singh C.S.B., Eyford B.A., Abraham T., Munro L., Choi K.B., Okon M., Vitalis T.Z., Gabathuler R., Lu C.J., Pfeifer C.G. (2021). Discovery of a Highly Conserved Peptide in the Iron Transporter Melanotransferrin that Traverses an Intact Blood Brain Barrier and Localizes in Neural Cells. Front. Neurosci..

[9] Kim K.A., Kim D., Kim J.H., Shin Y.J., Kim E.S., Akram M., Kim E.H., Majid A., Baek S.H., Bae O.N. (2020). Autophagy-mediated occludin degradation contributes to blood-brain barrier disruption during ischemia in bEnd.3 brain endothelial cells and rat ischemic stroke models. Fluids Barriers CNS.

[10] McCarthy R.C., Kosman D.J. (2015). Mechanisms and regulation of iron trafficking across the capillary endothelial cells of the blood-brain barrier. Front. Mol. Neurosci..

[11] McCarthy R.C., Kosman D.J. (2015). Iron transport across the blood-brain barrier: Development, neurovascular regulation and cerebral amyloid angiopathy. Cell. Mol. Life Sci..

[12] Burkhart A., Skjørringe T., Johnsen K.B., Siupka P., Thomsen L.B., Nielsen M.S., Thomsen L.L., Moos T. (2016). Expression of Iron-Related Proteins at the Neurovascular Unit Supports Reduction and Reoxidation of Iron for Transport Through the Blood-Brain Barrier. Mol. Neurobiol..

[13] Haldar S., Tripathi A., Qian J., Beserra A., Suda S., McElwee M., Turner J., Hopfer U., Singh N. (2015). Prion protein promotes kidney iron uptake via its ferrireductase activity. J. Biol. Chem..

[14] Yanatori I., Kishi F. (2019). DMT1 and iron transport. Free. Radic. Biol. Med..

[15] Cheng H., Wang N., Ma X., Wang P., Dong W., Chen Z., Wu M., Wang Z., Wang L., Guan D. (2022). Spatial-temporal changes of iron deposition and iron metabolism after traumatic brain injury in mice. Front. Mol. Neurosci..

[16] Brissot P., Ropert M., Le Lan C., Loréal O. (2012). Non-transferrin bound iron: A key role in iron overload and iron toxicity. Biochim. Biophys. Acta.

[17] Ounjaijean S., Thephinlap C., Khansuwan U., Phisalapong C., Fucharoen S., Porter J.B., Srichairatanakool S. (2008). Effect of green tea on iron status and oxidative stress in iron-loaded rats. Med. Chem..

[18] McCarthy R.C., Sosa J.C., Gardeck A.M., Baez A.S., Lee C.H., Wessling-Resnick M. (2018). Inflammation-induced iron transport and metabolism by brain microglia. J. Biol. Chem..

[19] McCarthy R.C., Kosman D.J. (2012). Mechanistic analysis of iron accumulation by endothelial cells of the BBB. Biometals Int. J. Role Met. Ions Biol. Biochem. Med..

[20] Long H., Zhu W., Wei L., Zhao J. (2023). Iron homeostasis imbalance and ferroptosis in brain diseases. MedComm.

[21] Ma Y., Okazaki Y., Glass J. (2018). A fluorescent metal-sensor study provides evidence for iron transport by transcytosis in the intestinal epithelial cells. J. Clin. Biochem. Nutr..

[22] Rodriguez-Callejas J.D., Fuchs E., Perez-Cruz C. (2020). Increased oxidative stress, hyperphosphorylation of tau, and dystrophic microglia in the hippocampus of aged Tupaia belangeri. Glia.

[23] Ward R.J., Zucca F.A., Duyn J.H., Crichton R.R., Zecca L. (2014). The role of iron in brain ageing and neurodegenerative disorders. Lancet Neurol..

[24] Skjørringe T., Burkhart A., Johnsen K.B., Moos T. (2015). Divalent metal transporter 1 (DMT1) in the brain: Implications for a role in iron transport at the blood-brain barrier, and neuronal and glial pathology. Front. Mol. Neurosci..

[25] Johannesson T., Kristinsson J., Torsdottir G., Snaedal J. (2012). Ceruloplasmin (Cp) and iron in connection with Parkinson’s disease (PD) and Alzheimer’s disease (AD). Laeknabladid.

[26] Ashraf A., Jeandriens J., Parkes H.G., So P.W. (2020). Iron dyshomeostasis, lipid peroxidation and perturbed expression of cystine/glutamate antiporter in Alzheimer’s disease: Evidence of ferroptosis. Redox Biol..

[27] Wagers M.L., Starks A., Abul-Khoudoud M.O., Ahmed S.M., Alhamdani A.W., Ashley C., Bidros P.C., Bledsoe C.O., Bolton K.E., Capili J.G. (2023). An invertebrate model in examining the effect of acute ferric iron exposure on proprioceptive neurons. Comp. Biochem. Physiol..

[28] Zhang X., Surguladze N., Slagle-Webb B., Cozzi A., Connor J.R. (2006). Cellular iron status influences the functional relationship between microglia and oligodendrocytes. Glia.

[29] Baringer S.L., Simpson I.A., Connor J.R. (2023). Brain iron acquisition: An overview of homeostatic regulation and disease dysregulation. J. Neurochem..

[30] Todorich B., Zhang X., Connor J.R. (2011). H-ferritin is the major source of iron for oligodendrocytes. Glia.

[31] Chiou B., Lucassen E., Sather M., Kallianpur A., Connor J. (2018). Semaphorin4A and H-ferritin utilize Tim-1 on human oligodendrocytes: A novel neuro-immune axis. Glia.

[32] Salnikow K. (2021). Role of iron in cancer. Semin. Cancer Biol..

[33] Jhurry N.D., Chakrabarti M., McCormick S.P., Holmes-Hampton G.P., Lindahl P.A. (2012). Biophysical investigation of the ironome of human jurkat cells and mitochondria. Biochemistry.

[34] Chakraborti S., Chakrabarti P. (2019). Self-Assembly of Ferritin: Structure, Biological Function and Potential Applications in Nanotechnology. Adv. Exp. Med. Biol..

[35] Kraml P. (2017). The role of iron in the pathogenesis of atherosclerosis. Physiol. Res..

[36] Sing R., Shikha D., Goswami C. (2025). TRPM8 modulation alters uptake of Transferrin-mediated Fe^3+^, mitochondrial Fe^2+^ and intracellular Ca^2+^-levels in microglia. Neurochem. Int..

[37] Yu H., Liu F., Hu Y., Wan W., Liu Q., Zhou S., Zhang L., Li L., Huang F. (2025). Injectable ion-coordinated double-network conductive hydrogel for spinal cord injury repair. Front. Bioeng. Biotechnol..

[38] Knutson M.D., Oukka M., Koss L.M., Aydemir F., Wessling-Resnick M. (2005). Iron release from macrophages after erythrophagocytosis is up-regulated by ferroportin 1 overexpression and down-regulated by hepcidin. Proc. Natl. Acad. Sci. USA.

[39] Camaschella C., Pagani A., Silvestri L., Nai A. (2022). The mutual crosstalk between iron and erythropoiesis. Int. J. Hematol..

[40] Wang C.Y., Babitt J.L. (2019). Liver iron sensing and body iron homeostasis. Blood.

[41] Zhou Z.D., Tan E.K. (2017). Iron regulatory protein (IRP)-iron responsive element (IRE) signaling pathway in human neurodegenerative diseases. Mol. Neurodegener..

[42] Pantopoulos K. (2004). Iron metabolism and the IRE/IRP regulatory system: An update. Ann. N. Y. Acad. Sci..

[43] Zhang Y., Lu Y., Jin L. (2022). Iron Metabolism and Ferroptosis in Physiological and Pathological Pregnancy. Int. J. Mol. Sci..

[44] Philpott C.C., Protchenko O., Wang Y., Novoa-Aponte L., Leon-Torres A., Grounds S., Tietgens A.J. (2023). Iron-tracking strategies: Chaperones capture iron in the cytosolic labile iron pool. Front. Mol. Biosci..

[45] Zhou M.Y., Xie F., Zhao Y., Wang X., Sun Z.W., Qian L.J. (2025). Stress-induced regional characteristics of the blood-brain barrier. Brain Res. Bull..

[46] Menard C., Pfau M.L., Hodes G.E., Kana V., Wang V.X., Bouchard S., Takahashi A., Flanigan M.E., Aleyasin H., LeClair K.B. (2017). Social stress induces neurovascular pathology promoting depression. Nat. Neurosci..

[47] Qie X., Wen D., Guo H., Xu G., Liu S., Shen Q., Liu Y., Zhang W., Cong B., Ma C. (2017). Endoplasmic Reticulum Stress Mediates Methamphetamine-Induced Blood-Brain Barrier Damage. Front. Pharmacol..

[48] Dion-Albert L., Cadoret A., Doney E., Kaufmann F.N., Dudek K.A., Daigle B., Parise L.F., Cathomas F., Samba N., Hudson N. (2022). Vascular and blood-brain barrier-related changes underlie stress responses and resilience in female mice and depression in human tissue. Nat. Commun..

[49] van Vliet E.A., Ndode-Ekane X.E., Lehto L.J., Gorter J.A., Andrade P., Aronica E., Gröhn O., Pitkänen A. (2020). Long-lasting blood-brain barrier dysfunction and neuroinflammation after traumatic brain injury. Neurobiol. Dis..

[50] Jamrus P., Pholngam N., Kiatpakdee B., Viwatpinyo K., Vadolas J., Ngampramuan S., Svasti S., Chaichompoo P. (2025). Iron induces blood-brain barrier alteration contributing to cognitive impairment in β-thalassaemia mice. Sci. Rep..

[51] Zhai R.F., Liu Q. (2024). Baicalin alleviates intestinal ischemia-reperfusion injury by regulating ferroptosis mediated by nuclear factor E2-related factor 2/Glutathione peroxidase 4 signaling pathway. J. Physiol. Pharmacol..

[52] Almutairi M.M., Gong C., Xu Y.G., Chang Y., Shi H. (2016). Factors controlling permeability of the blood-brain barrier. Cell. Mol. Life Sci..

[53] Rust C., Malan-Muller S., van den Heuvel L.L., Tonge D., Seedat S., Pretorius E., Hemmings S.M.J. (2023). Platelets bridging the gap between gut dysbiosis and neuroinflammation in stress-linked disorders: A narrative review. J. Neuroimmunol..

[54] Yang Y., Lv M., Liu R., Yu P., Shen Z., Bai D., Zhao P., Yang J., Tang X., Yang H. (2025). Tungsten-based polyoxometalate nanoclusters as ferroptosis inhibitors modulating S100A8/A9-mediated iron metabolism pathway for managing intracerebral haemorrhage. J. Nanobiotechnol..

[55] Delgado-Martín S., Martínez-Ruiz A. (2024). The role of ferroptosis as a regulator of oxidative stress in the pathogenesis of ischemic stroke. FEBS Lett..

[56] Cheng Y., Qu W., Li J., Jia B., Song Y., Wang L., Rui T., Li Q., Luo C. (2022). Ferristatin II, an Iron Uptake Inhibitor, Exerts Neuroprotection against Traumatic Brain Injury via Suppressing Ferroptosis. ACS Chem. Neurosci..

[57] Kawabata H. (2019). Transferrin and transferrin receptors update. Free. Radic. Biol. Med..

[58] Ji C., Steimle B.L., Bailey D.K., Kosman D.J. (2018). The Ferroxidase Hephaestin But Not Amyloid Precursor Protein is Required for Ferroportin-Supported Iron Efflux in Primary Hippocampal Neurons. Cell. Mol. Neurobiol..

[59] Loke S.Y., Siddiqi N.J., Alhomida A.S., Kim H.C., Ong W.Y. (2013). Expression and localization of duodenal cytochrome b in the rat hippocampus after kainate-induced excitotoxicity. Neuroscience.

[60] Pérez-Núñez R., González M.F., Avalos A.M., Leyton L. (2025). Impacts of PI3K/protein kinase B pathway activation in reactive astrocytes: From detrimental effects to protective functions. Neural Regen. Res..

[61] Guo J.J., Yue F., Song D.Y., Bousset L., Liang X., Tang J., Yuan L., Li W., Melki R., Tang Y. (2021). Intranasal administration of α-synuclein preformed fibrils triggers microglial iron deposition in the substantia nigra of Macaca fascicularis. Cell Death Dis..

[62] Xu M., Li Y., Meng D., Zhang D., Wang B., Xie J., Wang J. (2022). 6-Hydroxydopamine Induces Abnormal Iron Sequestration in BV2 Microglia by Activating Iron Regulatory Protein 1 and Inhibiting Hepcidin Release. Biomolecules.

[63] Pandur E., Tamási K., Pap R., Varga E., Miseta A., Sipos K. (2019). Fractalkine Induces Hepcidin Expression of BV-2 Microglia and Causes Iron Accumulation in SH-SY5Y Cells. Cell. Mol. Neurobiol..

[64] Lee S., Martinez-Valbuena I., Lang A.E., Kovacs G.G. (2024). Cellular iron deposition patterns predict clinical subtypes of multiple system atrophy. Neurobiol. Dis..

[65] Lu L.N., Qian Z.M., Wu K.C., Yung W.H., Ke Y. (2017). Expression of Iron Transporters and Pathological Hallmarks of Parkinson’s and Alzheimer’s Diseases in the Brain of Young, Adult, and Aged Rats. Mol. Neurobiol..

[66] Lee D.G., Park J., Lee H.S., Lee S.R., Lee D.S. (2016). Iron overload-induced calcium signals modulate mitochondrial fragmentation in HT-22 hippocampal neuron cells. Toxicology.

[67] Franco R., Navarro G., Martínez-Pinilla E. (2019). Antioxidant Defense Mechanisms in Erythrocytes and in the Central Nervous System. Antioxidants.

[68] Lucius R., Sievers J. (1996). Postnatal retinal ganglion cells in vitro: Protection against reactive oxygen species (ROS)-induced axonal degeneration by cocultured astrocytes. Brain Res..

[69] Dyer R.G., Burnet F. (1976). Effects of ferrous ions on preoptic area neurones and luteinizing hormone secretion in the rat. J. Endocrinol..

[70] Quiles Del Rey M., Mancias J.D. (2019). NCOA4-Mediated Ferritinophagy: A Potential Link to Neurodegeneration. Front. Neurosci..

[71] Hausmann A., Lee J., Pantopoulos K. (2011). Redox control of iron regulatory protein 2 stability. FEBS Lett..

[72] Jayakumar D., KK S.N., Chandrasekar N., Rajkumar A., Panchalingam G., Ravikumar V.C., Periandavan K. (2025). Nuclear factor, erythroid 2 like 2 (NRF2)-mediated disruption of iron homeostasis drives myocardial infarction progression. Br. J. Pharmacol..

[73] Jiang S., Guo T., Guo S., Gao J., Ni Y., Ma W., Zhao R. (2021). Chronic Variable Stress Induces Hepatic Fe(II) Deposition by Up-Regulating ZIP14 Expression via miR-181 Family Pathway in Rats. Biology.

[74] Piloni N.E., Perazzo J.C., Fernandez V., Videla L.A., Puntarulo S. (2016). Sub-chronic iron overload triggers oxidative stress development in rat brain: Implications for cell protection. Biometals Int. J. Role Met. Ions Biol. Biochem. Med..

[75] Wang L., Wang W., Zhao M., Ma L., Li M. (2008). Psychological stress induces dysregulation of iron metabolism in rat brain. Neuroscience.

[76] Wojciechowska M., Wisniewski O.W., Kolodziejski P., Krauss H. (2021). Role of hepcidin in physiology and pathophysiology. Emerging experimental and clinical evidence. J. Physiol. Pharmacol..

[77] Tajes M., Díez-López C., Enjuanes C., Moliner P., Ferreiro J.L., Garay A., Jiménez-Marrero S., Yun S., Sosa S.G., Alcoberro L. (2021). Neurohormonal activation induces intracellular iron deficiency and mitochondrial dysfunction in cardiac cells. Cell Biosci..

[78] Yan Y., Wang Z., Wei W., Yang Z., Guo L., Wang Z., Wei X. (2023). Correlation of brain iron deposition and freezing of gait in Parkinson’s disease: A cross-sectional study. Quant. Imaging Med. Surg..

[79] Wang L., Wang H., Li L., Li W., Dong X., Li M., Lv L. (2010). Corticosterone induces dysregulation of iron metabolism in hippocampal neurons in vitro. Biol. Trace Elem. Res..

[80] Reid B.M., Georgieff M.K. (2023). The Interaction between Psychological Stress and Iron Status on Early-Life Neurodevelopmental Outcomes. Nutrients.

[81] Mao C., Lei G., Horbath A., Gan B. (2022). Assessment of lipid peroxidation in irradiated cells. Methods Cell Biol..

[82] Yang D., Gong G., Song J., Chen J., Wang S., Li J., Wang G. (2025). Ferroptosis-mediated osteoclast-osteoblast crosstalk: Signaling pathways governing bone remodeling in osteoporosis. J. Orthop. Surg. Res..

[83] Lan J., Zhou Y., Deng L., Liu H., Hong W., Jing R., Zheng J. (2025). Epigenetic Regulation of Ferroptosis by the KDM3A/BRPF1 Transcriptional Axis Confers Neuroprotection in Postpartum Depression mouse model. J. Affect. Disord..

[84] Ma X., Shi W., Wang Z., Li S., Ma R., Zhu W., Wu L., Feng X., Cong B., Li Y. (2025). Butyric acid and valeric acid attenuate stress-induced ferroptosis and depressive-like behaviors by suppressing hippocampal neuroinflammation. J. Transl. Med..

[85] Gao J., Song X., Feng Y., Wu L., Ding Z., Qi S., Yu M., Wu R., Zheng X., Qin Y. (2024). Electroacupuncture ameliorates depression-like behaviors in rats with post-stroke depression by inhibiting ferroptosis in the prefrontal cortex. Front. Neurosci..

[86] Fernández-Mendívil C., Luengo E., Trigo-Alonso P., García-Magro N., Negredo P., López M.G. (2021). Protective role of microglial HO-1 blockade in aging: Implication of iron metabolism. Redox Biol..

[87] Kvarta M.D., Bradbrook K.E., Dantrassy H.M., Bailey A.M., Thompson S.M. (2015). Corticosterone mediates the synaptic and behavioral effects of chronic stress at rat hippocampal temporoammonic synapses. J. Neurophysiol..

[88] Feng Z., Min L., Chen H., Deng W., Tan M., Liu H., Hou J. (2021). Iron overload in the motor cortex induces neuronal ferroptosis following spinal cord injury. Redox Biol..

[89] Wang J., Zhu Q., Wang Y., Peng J., Shao L., Li X. (2022). Irisin protects against sepsis-associated encephalopathy by suppressing ferroptosis via activation of the Nrf2/GPX4 signal axis. Free. Radic. Biol. Med..

[90] Huang L., Zhang Y., Zhao L., Chen Q., Li L. (2022). Ferrostatin-1 Polarizes Microglial Cells Toward M2 Phenotype to Alleviate Inflammation After Intracerebral Hemorrhage. Neurocritical Care.

[91] Yao M.Y., Liu T., Zhang L., Wang M.J., Yang Y., Gao J. (2021). Role of ferroptosis in neurological diseases. Neurosci. Lett..

[92] Möller H.E., Bossoni L., Connor J.R., Crichton R.R., Does M.D., Ward R.J., Zecca L., Zucca F.A., Ronen I. (2019). Iron, Myelin, and the Brain: Neuroimaging Meets Neurobiology. Trends Neurosci..

[93] Thirupathi A., Chang Y.Z. (2019). Brain Iron Metabolism and CNS Diseases. Adv. Exp. Med. Biol..

[94] Fanzani A., Poli M. (2017). Iron, Oxidative Damage and Ferroptosis in Rhabdomyosarcoma. Int. J. Mol. Sci..

[95] Han H., Zhang G., Zhang X., Zhao Q. (2024). Nrf2-mediated ferroptosis inhibition: A novel approach for managing inflammatory diseases. Inflammopharmacology.

[96] Del Vecchio L., Girelli D., Vinchi F., Cozzolino M., Elliott S., Mark P.B., Valenti L., Qian C., Guo Q., Qian Z.M. (2024). Iron biology. Nephrol. Dial. Transplant. Off. Publ. Eur. Dial. Transpl. Assoc.—Eur. Ren. Assoc..

[97] Jiang Z., Zhou J., Deng J., Li L., Wang R., Han Y., Zhou J., Tao R., Peng L., Wang D. (2023). Emerging roles of ferroptosis-related miRNAs in tumor metastasis. Cell Death Discov..

[98] An H., Zeng X., Niu T., Li G., Yang J., Zheng L., Zhou W., Liu H., Zhang M., Huang D. (2018). Quantifying iron deposition within the substantia nigra of Parkinson’s disease by quantitative susceptibility mapping. J. Neurol. Sci..

[99] Sofic E., Riederer P., Heinsen H., Beckmann H., Reynolds G.P., Hebenstreit G., Youdim M.B. (1988). Increased iron (III) and total iron content in post mortem substantia nigra of parkinsonian brain. J. Neural Transm..

[100] Zhang J., Zhang Y., Wang J., Cai P., Luo C., Qian Z., Dai Y., Feng H. (2010). Characterizing iron deposition in Parkinson’s disease using susceptibility-weighted imaging: An in vivo MR study. Brain Res..

[101] Song N., Wang J., Jiang H., Xie J. (2018). Astroglial and microglial contributions to iron metabolism disturbance in Parkinson’s disease. Biochim. Biophys. Acta Mol. Basis Dis..

[102] Jia W., Xu H., Du X., Jiang H., Xie J. (2015). Ndfip1 attenuated 6-OHDA-induced iron accumulation via regulating the degradation of DMT1. Neurobiol. Aging.

[103] Dos Santos Cardoso F., Arida R.M., Alves da Silva E., de Oliveira A.C.R., Ferreira Dos Santos W., Lopes-Martins R.Á.B., Gonzalez-Lima F., Coimbra N.C., Gomes da Silva S. (2025). Effects of Chronic Photobiomodulation with Transcranial Near-Infrared Laser on Seizure Frequency and Brain Metabolomics of Rats with Pilocarpine-Induced Seizures. Mol. Neurobiol..

[104] Szu J.I., Binder D.K. (2022). Mechanisms Underlying Aquaporin-4 Subcellular Mislocalization in Epilepsy. Front. Cell. Neurosci..

[105] Wang Y., Tan B., Wang Y., Chen Z. (2021). Cholinergic Signaling, Neural Excitability, and Epilepsy. Molecules.

[106] Zimmer T.S., David B., Broekaart D.W.M., Schidlowski M., Ruffolo G., Korotkov A., van der Wel N.N., van Rijen P.C., Mühlebner A., van Hecke W. (2021). Seizure-mediated iron accumulation and dysregulated iron metabolism after status epilepticus and in temporal lobe epilepsy. Acta Neuropathol..

[107] Duan L., Yin X., Meng H., Fang X., Min J., Wang F. (2020). Progress on epigenetic regulation of iron homeostasis. J. Zhejiang Univ. Med. Sci..

[108] Roggan M.D., Kronenberg J., Wollert E., Hoffmann S., Nisar H., Konda B., Diegeler S., Liemersdorf C., Hellweg C.E. (2023). Unraveling astrocyte behavior in the space brain: Radiation response of primary astrocytes. Front. Public Health.

[109] Podvin S., Reardon H.T., Yin K., Mosier C., Hook V. (2019). Multiple clinical features of Huntington’s disease correlate with mutant HTT gene CAG repeat lengths and neurodegeneration. J. Neurol..

[110] Tong H., Yang T., Liu L., Li C., Sun Y., Jia Q., Qin Y., Chen L., Zhao X., Zhou G. (2023). Aberrant splicing of mutant huntingtin in Huntington’s disease knock-in pigs. Neurobiol. Dis..

[111] van Bergen J.M., Hua J., Unschuld P.G., Lim I.A., Jones C.K., Margolis R.L., Ross C.A., van Zijl P.C., Li X. (2016). Quantitative Susceptibility Mapping Suggests Altered Brain Iron in Premanifest Huntington Disease. Am. J. Neuroradiol..

[112] Chen J., Marks E., Lai B., Zhang Z., Duce J.A., Lam L.Q., Volitakis I., Bush A.I., Hersch S., Fox J.H. (2013). Iron accumulates in Huntington’s disease neurons: Protection by deferoxamine. PLoS ONE.

[113] Dumas E.M., Versluis M.J., van den Bogaard S.J., van Osch M.J., Hart E.P., van Roon-Mom W.M., van Buchem M.A., Webb A.G., van der Grond J., Roos R.A. (2012). Elevated brain iron is independent from atrophy in Huntington’s Disease. NeuroImage.

[114] Liang W., Zhou B., Miao Z., Liu X., Liu S. (2024). Abnormality in Peripheral and Brain Iron Contents and the Relationship with Grey Matter Volumes in Major Depressive Disorder. Nutrients.

[115] Yao S., Zhong Y., Xu Y., Qin J., Zhang N., Zhu X., Li Y. (2017). Quantitative Susceptibility Mapping Reveals an Association between Brain Iron Load and Depression Severity. Front. Hum. Neurosci..

[116] Duan X., Xie Y., Zhu X., Chen L., Li F., Feng G., Li L. (2022). Quantitative Susceptibility Mapping of Brain Iron Deposition in Patients With Recurrent Depression. Psychiatry Investig..

[117] Zhao M., Yu Z., Zhang Y., Huang X., Hou J., Zhao Y., Luo W., Chen L., Ou L., Li H. (2016). Iron-induced neuronal damage in a rat model of post-traumatic stress disorder. Neuroscience.

[118] Islam M.R., Ahmed M.U., Mitu S.A., Islam M.S., Rahman G.K., Qusar M.M., Hasnat A. (2013). Comparative analysis of serum zinc, copper, manganese, iron, calcium, and magnesium level and complexity of interelement relations in generalized anxiety disorder patients. Biol. Trace Elem. Res..

[119] Duarte-Silva E., Maes M., Alves Peixoto C. (2025). Iron metabolism dysfunction in neuropsychiatric disorders: Implications for therapeutic intervention. Behav. Brain Res..

[120] Cuesta M.J., Lecumberri P., Moreno-Izco L., López-Ilundain J.M., Ribeiro M., Cabada T., Lorente-Omeñaca R., de Erausquin G., García-Martí G., Sanjuan J. (2021). Motor abnormalities and basal ganglia in first-episode psychosis (FEP). Psychol. Med..

[121] Xu M., Guo Y., Cheng J., Xue K., Yang M., Song X., Feng Y., Cheng J. (2021). Brain iron assessment in patients with First-episode schizophrenia using quantitative susceptibility mapping. NeuroImage Clin..

[122] Mandal P.K., Gaur S., Roy R.G., Samkaria A., Ingole R., Goel A. (2022). Schizophrenia, Bipolar and Major Depressive Disorders: Overview of Clinical Features, Neurotransmitter Alterations, Pharmacological Interventions, and Impact of Oxidative Stress in the Disease Process. ACS Chem. Neurosci..

[123] Keleş Altun İ., Atagün M., Erdoğan A., Oymak Yenilmez D., Yusifova A., Şenat A., Erel Ö. (2021). Serum hepcidin/ferroportin levels in bipolar disorder and schizophrenia. J. Trace Elem. Med. Biol. Organ Soc. Miner. Trace Elem. (GMS).

[124] Reid B.M. (2024). Early life stress and iron metabolism in developmental psychoneuroimmunology. Brain Behav. Immun.—Health.

[125] Vuuren M.J.V., Nell T.A., Carr J.A., Kell D.B., Pretorius E. (2020). Iron Dysregulation and Inflammagens Related to Oral and Gut Health Are Central to the Development of Parkinson’s Disease. Biomolecules.

[126] Hu S., Yang B., Li B., Fan Q., Wu T., Li S., Wang D., Yang T., Song Z. (2024). RNA-Seq Analysis Reveals Potential Neuroprotective Mechanisms of Pachymic Acid Toward Iron-Induced Oxidative Stress and Cell Death. Cell Transplant..

[127] Kukuia K.K.E., Torbi J., Amoateng P., Adutwum-Ofosu K.K., Koomson A.E., Appiah F., Tagoe T.A., Mensah J.A., Ameyaw E.O., Adi-Dako O. (2022). Gestational iron supplementation reverses depressive-like behavior in post-partum Sprague Dawley rats: Evidence from behavioral and neurohistological studies. IBRO Neurosci. Rep..

[128] Liu X., Luo Q., Zhao Y., Ren P., Jin Y., Zhou J. (2025). The Ferroptosis-Mitochondrial Axis in Depression: Unraveling the Feedforward Loop of Oxidative Stress, Metabolic Homeostasis Dysregulation, and Neuroinflammation. Antioxidants.

[129] Maniscalchi A., Benzi Juncos O.N., Conde M.A., Funk M.I., Fermento M.E., Facchinetti M.M., Curino A.C., Uranga R.M., Alza N.P., Salvador G.A. (2024). New insights on neurodegeneration triggered by iron accumulation: Intersections with neutral lipid metabolism, ferroptosis, and motor impairment. Redox Biol..

[130] Marupudi N., Xiong M.P. (2024). Genetic Targets and Applications of Iron Chelators for Neurodegeneration with Brain Iron Accumulation. ACS Bio Med Chem Au.

[131] Nuñez M.T., Chana-Cuevas P. (2018). New Perspectives in Iron Chelation Therapy for the Treatment of Neurodegenerative Diseases. Pharmaceuticals.

[132] Botzenhardt S., Li N., Chan E.W., Sing C.W., Wong I.C., Neubert A. (2017). Safety profiles of iron chelators in young patients with haemoglobinopathies. Eur. J. Haematol..

[133] Tanous O., Azulay Y., Halevy R., Dujovny T., Swartz N., Colodner R., Koren A., Levin C. (2021). Renal function in β-thalassemia major patients treated with two different iron-chelation regimes. BMC Nephrol..

[134] Toliyat T., Jorjani M., Khorasanirad Z. (2009). An extended-release formulation of desferrioxamine for subcutaneous administration. Drug Deliv..

[135] Yeo J.H., Begam N., Leow W.T., Goh J.X., Zhong Y., Cai Y., Kwa A.L. (2024). Ironing out Persisters? Revisiting the Iron Chelation Strategy to Target Planktonic Bacterial Persisters Harboured in Carbapenem-Resistant Escherichia coli. Microorganisms.

[136] Salimi Z., Afsharinasab M., Rostami M., Eshaghi Milasi Y., Mousavi Ezmareh S.F., Sakhaei F., Mohammad-Sadeghipour M., Rasooli Manesh S.M., Asemi Z. (2024). Iron chelators: As therapeutic agents in diseases. Ann. Med. Surg..

[137] Sridharan K., Sivaramakrishnan G. (2018). Efficacy and safety of iron chelators in thalassemia and sickle cell disease: A multiple treatment comparison network meta-analysis and trial sequential analysis. Expert Rev. Clin. Pharmacol..

[138] Dusek P., Schneider S.A., Aaseth J. (2016). Iron chelation in the treatment of neurodegenerative diseases. J. Trace Elem. Med. Biol. Organ Soc. Miner. Trace Elem. (GMS).

[139] Sandoval T.A., Salvagno C., Chae C.S., Awasthi D., Giovanelli P., Marin Falco M., Hwang S.M., Teran-Cabanillas E., Suominen L., Yamazaki T. (2024). Iron Chelation Therapy Elicits Innate Immune Control of Metastatic Ovarian Cancer. Cancer Discov..

[140] Lv Q., Lin J., Huang H., Ma B., Li W., Chen J., Wang M., Wang X., Fu G., Xiao Y. (2024). Nanosponge for Iron Chelation and Efflux: A Ferroptosis-Inhibiting Approach for Myocardial Infarction Therapy. Adv. Sci..

[141] Xu Y., Wang Y., An J., Sedgwick A.C., Li M., Xie J., Hu W., Kang J., Sen S., Steinbrueck A. (2022). 2D-ultrathin MXene/DOXjade platform for iron chelation chemo-photothermal therapy. Bioact. Mater..

[142] Singh R., Panghal A., Jadhav K., Thakur A., Verma R.K., Singh C., Goyal M., Kumar J., Namdeo A.G. (2024). Recent Advances in Targeting Transition Metals (Copper, Iron, and Zinc) in Alzheimer’s Disease. Mol. Neurobiol..

[143] Li Y., Xiao D., Wang X. (2022). The emerging roles of ferroptosis in cells of the central nervous system. Front. Neurosci..

[144] Guo X., Qi X., Li H., Duan Z., Wei Y., Zhang F., Tian M., Ma L., You C. (2019). Deferoxamine Alleviates Iron Overload and Brain Injury in a Rat Model of Brainstem Hemorrhage. World Neurosurg..

[145] Zhu Y., Wang B., Tao K., Yang H., Wang Y., Zhou T., Yang Y., Yuan L., Liu X., Duan Y. (2017). Iron accumulation and microglia activation contribute to substantia nigra hyperechogenicity in the 6-OHDA-induced rat model of Parkinson’s disease. Park. Relat. Disord..

[146] Yuan Y., He Q., Yang X., Flores J.J., Huang L., Luo X., Zhang X., Zhang Z., Li R., Gu L. (2024). Mitochondrial ferritin upregulation reduced oxidative stress and blood-brain-barrier disruption by maintaining cellular iron homeostasis in a neonatal rat model of germinal matrix hemorrhage. Exp. Neurol..

[147] Goswami D., Vitorino H.A., Alta R.Y., Silvestre D.M., Nomura C.S., Machini M.T., Espósito B.P. (2015). Deferasirox-TAT(47-57) peptide conjugate as a water soluble, bifunctional iron chelator with potential use in neuromedicine. Biometals Int. J. Role Met. Ions Biol. Biochem. Med..

[148] Ren S., Han S., Wang L., Huang Y., Wu J., Wu G. (2022). Minimally Invasive Surgery for ICH Evacuation Combined with Deferoxamine Treatment Increased Perihematomal Claudin-5 and ZO-1 Expression Levels and Decreased BBB Permeability in Rabbits. Front. Neurol..

[149] Li X., Jiang X.W., Chu H.X., Zhao Q.C., Ding H.W., Cai C.H. (2021). Neuroprotective effects of kukoamine A on 6-OHDA-induced Parkinson’s model through apoptosis and iron accumulation inhibition. Chin. Herb. Med..

[150] Li Y., Ruan X., Sun M., Yuan M., Song J., Zhou Z., Li H., Ma Y., Mi W., Zhang X. (2024). Iron deposition participates in LPS-induced cognitive impairment by promoting neuroinflammation and ferroptosis in mice. Exp. Neurol..

[151] Li B., Espósito B.P., Wang S., Zhang J., Xu M., Zhang S., Zhang Z., Liu S. (2019). Desferrioxamine-caffeine shows improved efficacy in chelating iron and depleting cancer stem cells. J. Trace Elem. Med. Biol. Organ Soc. Miner. Trace Elem. (GMS).

[152] Elalfy M., Wali Y.A., Qari M., Al Damanhouri G., Al-Tonbary Y., Yazman D., Al Hawsawi Z., Karakas Z., Kilinc Y., Yesilipek M.A. (2014). Deviating from safety guidelines during deferiprone therapy in clinical practice may not be associated with higher risk of agranulocytosis. Pediatr. Blood Cancer.

[153] Fucile C., Mattioli F., Marini V., Gregori M., Sonzogni A., Martelli A., Maximova N. (2018). What is known about deferasirox chelation therapy in pediatric HSCT recipients: Two case reports of metabolic acidosis. Ther. Clin. Risk Manag..

